# Theta and Gamma Transcranial Alternating Current Stimulation (tACS) Modulate Mandarin Consonant and Lexical Tone Perception

**DOI:** 10.1162/NOL.a.285

**Published:** 2026-09-14

**Authors:** Yaxuan Wang, Keke Yu, Shuqi Yin, Baishen Liang, Ruiming Wang

**Affiliations:** Philosophy and Social Science Laboratory of Reading and Development in Children and Adolescents, Ministry of Education, South China Normal University; Center for Studies of Psychological Application, School of Psychology, South China Normal University, Guangzhou, China; Department of Neurology, Duke University, Durham, NC, USA

**Keywords:** consonant, lexical tone, Mandarin, theta and gamma activity, transcranial alternating current stimulation (tACS)

## Abstract

A theta/gamma neural activity mechanism has been postulated to explain the auditory sampling of hierarchical syllable-phoneme structure with corresponding speech rates or linguistic hierarchies (theta for syllable and gamma for phoneme). Yet, whether such a mechanism is generalizable to Mandarin Chinese, a tonal language, where lexical tones are at syllable length but function as phonemic cues, is unclear. In this study, we applied transcranial alternating current stimulation (tACS) with theta or gamma currents over bilateral temporal areas in healthy Mandarin-speaking participants during a consonant or tone identification task in quiet or noisy environments. Results showed that theta tACS impaired both consonant and tone identification in quiet and prolonged reaction times across tasks and environments. Moreover, it reduced the psychometric slope for consonant identification in quiet, indicating decreased perceptual sensitivity and less distinct category boundaries. Gamma tACS, however, only delayed tone identification in quiet. Besides, both theta and gamma tACS modulated perceptual decision-making parameters in quiet, leading to increased boundary thresholds (cautiousness of decision) and altered response biases in the perceptual decision of both consonants and tones, as evidenced by the hierarchical drift-diffusion model. These findings indicate that the perception of Mandarin consonants and lexical tones is causally supported by syllable-length theta activity, presumably reflecting syllable-level holistic representations, regardless of environment. Gamma activity, however, is presumably engaged in supporting fast-changing and fine-grained acoustic features in a modulatory manner. Theoretical explanations from historical linguistics and implications for clinical or educational applications are also provided.

## INTRODUCTION

Spoken Mandarin relies on the perception of syllables, which integrate phonemic features such as consonants and lexical tones (Chen & Zhang, 2025). This requires the auditory neural system to sample speech across multiple timescales. Neural activity at different frequency bands is believed to play a key role in tracking and sampling hierarchical speech signals (Giraud & Poeppel, 2012; Meyer, 2018), forming spectrotemporal receptive fields (STRFs) with varying sampling window lengths (Aertsen & Johannesma, 1981; Hasson et al., 2008). Especially, neural activity in the theta and gamma bands is shown to support speech sampling by aligning with distinct acoustic structures.

Theta activity (4–8 Hz) has been found to entrain to the syllabic rhythm of speech (Oganian et al., 2023), thereby supporting temporal parsing at the syllable level, and to contribute to the processing of slower suprasegmental structures, including phrasal boundaries and prosodic cues such as intonation (Teoh et al., 2019), as well as lexical tones (Meyer, 2018; Wang et al., 2017). In Mandarin, which has syllable-level lexical tones as distinguishing features for meanings (Duanmu, 2007), theta activity with a period at the syllabic length also plays a key role by adaptively tracking syllabic speech rhythms to optimize perception (He et al., 2023; Ni et al., 2023). Evidence from event-related potential (ERP; Zou et al., 2020) and articulatory phonetics (Kang & Xu, 2024; Liu & Xu, 2023) studies further suggests that lexical tones and phonemes (e.g., consonants or vowels) are processed in temporal synchrony, indicating that syllable perception in Mandarin depends on the coordinated timing of their linguistic components.

Complementing this, gamma activity (40 Hz) is involved in processing fine-grained phonemic features varying rapidly in time, including formant transitions (Fukuda et al., 2010) and voice onset times (Rufener, Oechslin, et al., 2016), which are crucial for differentiating meanings (e.g., “bear” vs. “pear”; Sammler et al., 2015). Besides, recent transcranial alternating current stimulation (tACS) studies modulating neural activity (Ghiani et al., 2021; Herrmann et al., 2016) have shown that theta and gamma stimulation selectively perturb the neural tracking of syllable rhythms (Zoefel et al., 2018) and consonant discrimination (Rufener, Oechslin, et al., 2016), respectively. In brief, theta activity supports global syllabic integration, whereas gamma activity resolves local phonetic features (Lizarazu et al., 2019; Morillon et al., 2012).

In addition, such a global-local theta/gamma sampling mechanism can be modulated in adverse listening conditions (Kegler & Reichenbach, 2021; Keshavarzi et al., 2020). Electroencephalogram (EEG) studies showed that theta activity is enhanced in speech-in-noise tasks, whereas gamma activity is maintained under clear speech conditions for fine-grained structure analysis (Yellamsetty & Bidelman, 2018).

Nevertheless, as most studies focused on non-tonal languages, it remains unclear whether and how theta/gamma sampling applies to tonal languages such as Mandarin Chinese (Huang & Liao, 2017). Particularly, Mandarin Chinese is a tonal language where lexical tones are at syllabic length, yet function as phonemes to determine meanings (i.e., “tonemes”) (Chao, 1968; Liang & Du, 2018). Moreover, historical linguistics has further confirmed the close links between lexical tones and consonantal phonemes of the onset. This is representative of the general *tonogenesis* phenomenon in tonal languages, where tonal contrasts developed from earlier non-tonal contrasts. For instance, as Old Chinese evolved into Middle and then Modern Chinese, the loss of the voicing contrast triggered a well-documented tonal split known as ‘*pingfen yinyang*’ (平分阴阳): syllables were grouped into the unaspirated onset in Tone 1, and the aspirated onset in Tone 2, respectively (Michaud & Sands, 2020). Therefore, given the functional and historical links between lexical tone and consonants, the neural processing mechanisms for the perception of linguistic features in tonal languages may be different from those in non-tonal languages, where distinct sampling mechanisms exist for both segmental and suprasegmental elements. Particularly, as lexical tones and consonants can have the same origins, for native speakers, gamma activity with short (segmental level) receptive fields may also sample longer (suprasegmental level) lexical tones; or, on the other hand, as lexical tones and consonants may share overlapped representations (presumably in an integrated embedded syllable format) in the perceptual space, theta activity with wider (syllable level) receptive fields may be required.

**Figure 1.**
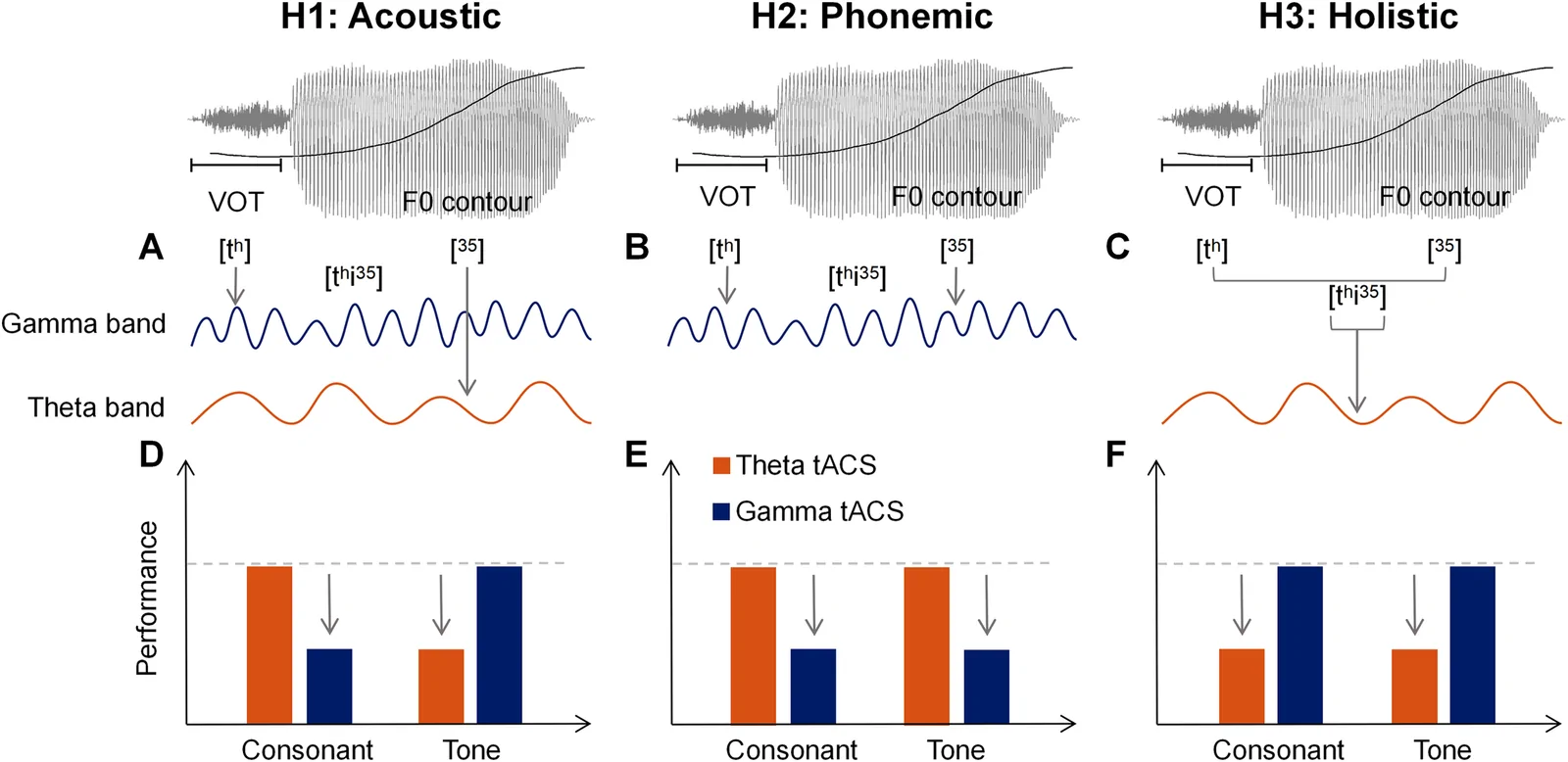
Three proposed hypotheses of Mandarin syllable perception and the predicted effects of tACS. The acoustic structure of a sample Mandarin syllable (e.g., “tí”, [t^h^i^35^]), showing the voice onset time (VOT) and the fundamental frequency (F0) contour. (A–C) Schematic illustrations of the gamma and theta oscillatory neural mechanisms proposed by each hypothesis to process the syllable. (D–F) Predicted patterns of performance modulation on consonant and tone identification tasks under theta and gamma tACS, relative to a sham stimulation baseline (gray dotted horizontal lines). Lower performance was expected when stimulation interfered with the neural activity most relevant for the target task. H1: Acoustic: Hypothesis 1 (the Acoustic Hypothesis); H2: Phonemic: Hypothesis 2 (the Phonemic Hypothesis); H3: Holistic: Hypothesis 3 (the Holistic Hypothesis).

Therefore, an unresolved question is whether, and how, syllable perception in Mandarin relies on frequency-specific neural activity, and whether the contributions of these frequency bands are altered in adverse listening conditions (e.g., when background noise is added). Based on previous work, we propose three competing hypotheses. Hypothesis 1, the Acoustic Hypothesis (Figure 1A), posits that theta and gamma activity sample suprasegmental (e.g., tones) and segmental (e.g., consonants) features, respectively, based on their acoustic length or linguistic hierarchy, resembling non-tonal languages. Hypothesis 2, the Phonemic Hypothesis (Figure 1B), suggests that both consonants and tones are encoded as phonemes, with gamma activity playing a dominant role. Hypothesis 3, the Holistic Hypothesis (Figure 1C), proposes that lexical tones and consonants share a perceptual space that is encoded as integrated syllabic units in perception, subserved by theta activity. The primary aim of the present study is to adjudicate among these three hypotheses by causally manipulating theta- and gamma-band activity during Mandarin syllable perception.

To adjudicate among these competing hypotheses, we employed an online, non-invasive brain stimulation paradigm with alternating-current theta and gamma band stimulation during Mandarin syllable perception. Participants performed a syllable identification task targeting both lexical tones and consonants, allowing us to assess the frequency-specific contributions of neural activity to the processing of both cues, respectively. Theta and gamma tACS, which have been shown to perturb the corresponding bands of neural activity (de Lara et al., 2018; Ghiani et al., 2021), were applied to bilateral temporal areas while participants were identifying the consonant or lexical tone of resynthesized, perceptually ambiguous syllables drawn from 5-step continua (i.e., [t]-[t^h^] and tone1-tone2). This design helps detect stimulation-induced changes in the perceptual sensitivity of speech categories (D’Ausilio et al., 2009; Liang et al., 2023a). Crucially, the tasks were conducted under both quiet and noisy listening conditions, as background noise is known to increase perceptual demands and may induce a re-organization of neural mechanisms supporting speech perception (Keshavarzi et al., 2020; Yellamsetty & Bidelman, 2018).

Behavioral performance was quantified using psychophysical curve slopes, points of subjective equity (PSE), and reaction times (RTs) for both lexical tone and consonant identification tasks, allowing us to assess how different frequencies stimulation modulate participants’ performance across auditory conditions. Previous studies have shown that tACS can either facilitate or impair speech perception depending on the stimulation protocol. Facilitative effects are more often observed when stimulation is temporally aligned with the speech envelopes or speech rhythms (e.g., Keshavarzi et al., 2020; Riecke et al., 2018), whereas fixed-frequency stimulation without such alignment has more commonly been associated with disruptive effects on speech processing and perceptual learning (e.g., Rufener, Zaehle, et al., 2016). Because stimulation in the present study was not phase-locked but instead temporally jittered relative to the speech stimuli, we expected it to interfere with endogenous neural dynamics supporting speech perception.

Therefore, under the Acoustic Hypothesis (Figure 1D), we predict that electrical stimulation at different frequencies will impair behavioral performance in the two tasks, respectively. Specifically, compared to sham stimulation, we predict that gamma tACS will preferentially alter slopes, PSE, and/or RT in the consonant identification task. In contrast, theta tACS will selectively modulate performance in the tone identification task. Under the Phonemic Hypothesis (Figure 1E), gamma tACS was expected to similarly affect changes in slopes, PSE, and/or RT in both tasks. We expect the effects of theta tACS to be smaller. Finally, under the Holistic Hypothesis (Figure 1F), we propose that theta tACS may generally impair behavioral performance across both tasks by altering slopes, PSE, and/or RT. We expect gamma tACS to have no significant effect on performance. In addition, if adverse auditory conditions increase reliance on slower temporal integration mechanisms for speech perception, the modulatory effects of theta-band stimulation should be more pronounced in noisy environments than in quiet ones.

## MATERIALS AND METHODS

### Participants

An a priori analysis was conducted using G*Power 3.1 (Faul et al., 2009) for a three-way repeated-measures ANOVA with factors of *Stimulus type* (theta vs. gamma tACS), *Task type* (tone vs. consonant identification), and *Environment* (quiet vs. noisy). Using Cohen’s *f* as the effect size measure, a medium effect size (*f* = 0.25) was assumed based on Cohen’s conventional criteria (Cohen, 2013), given the heterogeneity of prior tACS paradigms and reported effect size metrics. With *α* = 0.05 and power (1 − *β*) = 0.80, the required minimum sample size was estimated to be 29 participants. We initially recruited 35 adult native Mandarin speakers aged 18–25 years (7 male, mean age = 20.57 years, standard deviation [*SD*] = 1.93), all of whom were undergraduate or graduate students. Five participants were excluded due to task difficulties in the consonant identification task (*n* = 1) or ineligibility (*n* = 4) that participants or a family member has a neurological or psychological disorder, a history of epilepsy, or an auditory threshold higher than 25 dB in a pure-tone hearing test within the 0.25 to 8 kHz frequency range. The final sample consisted of 30 participants (6 male, mean age = 20.67 years, *SD* = 1.84). All participants were right-handed, as assessed by a modified version of the Edinburgh Handedness Inventory (mean laterality quotient = 101.09, *SD* = 20.86). In this modified version, the original response options were simplified into three categories: left hand (−2 points), both hands (0 points), and right hand (2 points), with scoring methods consistent with the original inventory (see Questionnaire 3 of the Supplementary Materials; Supporting Information can be found at https://doi.org/10.1162/NOL.a.285). Participants all had normal hearing, as measured using customized MATLAB scripts with a pair of Edifier MR4 Monitor Speakers at frequencies of 0.25, 0.5, 1, 2, 3, 4, 6, and 8 kHz (≤25 dB sound pressure level [SPL]) in a soundproof room. None of them had a self or family history of neurological, traumatic, or psychiatric disease. Four of the 30 participants had received formal musical training that had lasted longer than 2 years. All participants gave written informed consent before the experiment and were paid 40 yuan per hour. The experimental protocol (SCNU-PSY-2024-266) was approved by the Human Research Ethics Committee for Non-Clinical Faculties of the School of Psychology, South China Normal University.

### Acoustic Stimulii

**Figure 2.**
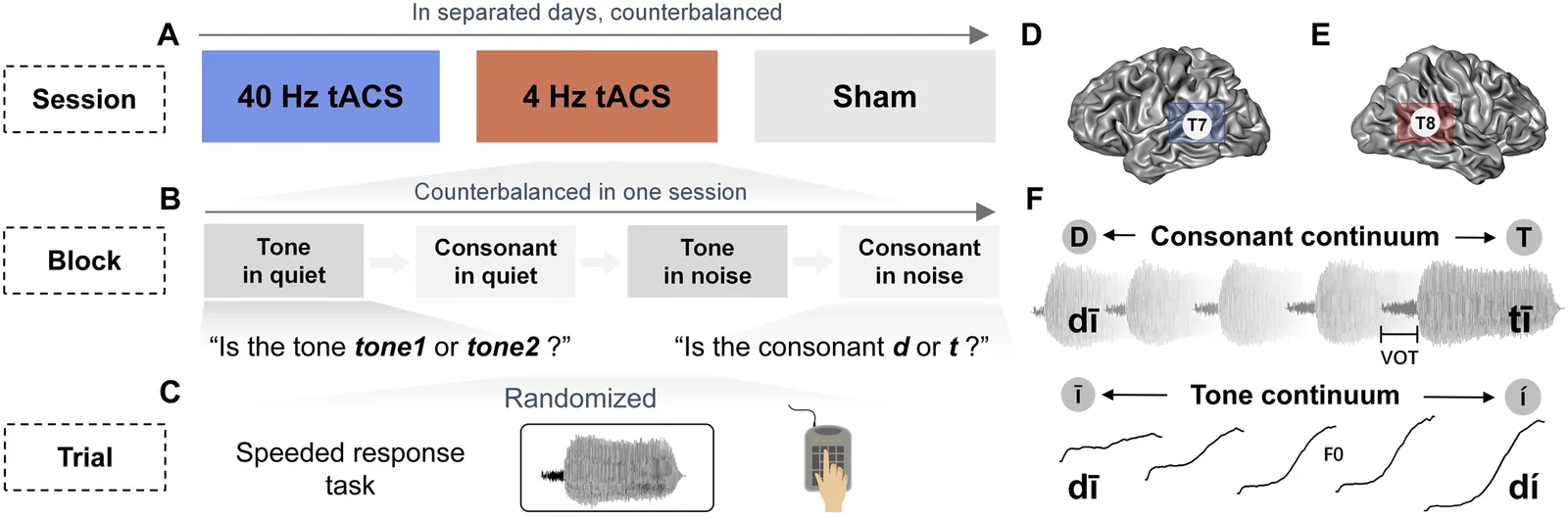
Experiment design, bilateral temporal montage, and stimulus continua. (A–C) Design of the experiment. (A) Participants received different patterns of transcranial alternating current stimulation (tACS) in different sessions with order counterbalanced: gamma band (40 Hz) tACS, theta band (4 Hz) tACS, and sham. (B) One session included four blocks with the order counterbalanced: tone and consonant identification tasks. (C) Participants made speeded decisions. The experimental stimuli consisted of four syllables created by crossing two tones (tone 1 [^55^] and tone 2 [^35^]) with two consonants ([t] and [t^h^]), resulting in four unique syllables: [ti^55^] (堤, riverbank), [ti^35^] (敌, enemy), [t^h^i^55^] (梯, ladder), and [t^h^i^35^] (题, title). (D–E) Bilateral temporal montage. Theta or gamma band tACS was applied to bilateral temporal areas by 5 × 7 cm sponge electrodes placed horizontally over T7 and T8 (10-20 EEG montage). (D) Left view. (E) Right view. Figures were adapted from the Brain Tutor 3D software (*Brain Tutor*, n.d.). Note that the polarities and values alternated over time. (F) Phonetic continua. Two types of five-step continua were synthesized. The consonant continuum (top) varied in voice onset time (VOT) from [t] to [t^h^]. The tone continuum (bottom) varied in fundamental frequency (F0) from Mandarin tone 1 (high level) to tone 2 (rising).

Syllables used in this experiment are four natural Mandarin monosyllables: [ti^55^], [ti^35^], [t^h^i^55^], and [t^h^i^35^], transcribed using both the International Phonetic Alphabet and Chao’s “tone-letters” system. Stimulus recording, annotation, and post-processing procedures were based on those described in Liang et al. (2023a). These corresponded to Mandarin syllables “堤 dī” (riverbank), “敌 dí” (enemy), “梯 tī” (ladder), and “题 tí” (title), respectively, in Pinyin. Participants were only exposed to the spoken syllables. No orthographic representations (Chinese characters) or explicit morphemic information were provided. Syllables were low-pass filtered at 5 kHz using Praat 6 algorithms and matched for average root-mean-square SPL. To account for acoustic features, the four syllables were denoised, re-synthesized, and pitch contours normalized using Praat 6. In the pretest of the experiment, individualized tone continua ([ti^55^] to [ti^35^]) and consonant continua ([ti^55^] to [t^h^i^55^]) were generated based on four natural syllables by stepwise adjustment of F0 and addition of aspiration noise, with each continuum consisting of five steps (Figure 2F). This approach ensured a range of categorical clarity across the stimuli, as categorically quiet speech has been shown to exhibit greater resistance to noise interference compared to ambiguous speech categories (Bidelman et al., 2020). Crucially, the phonetic continua allow for a comprehensive examination of perception under varying noise conditions. Midpoints in the continuum, rather than eliciting ceiling or floor effects, provide an optimal window to detect neuromodulation effects, as small perceptual biases induced by tACS can be more readily observed. This design thus enables a more precise assessment of neuro-modulatory effects across both quiet and noisy contexts.

The auditory stimuli were delivered using Edifier MR4 Monitor Speakers in stereo. A Deli DL333202 decibel meter (A-weighted) was employed to measure and calibrate the intensity levels of stimuli. In this experiment, each syllable was presented at a SPL of 65 dB through Psychtoolbox-3 in MATLAB R2016a.

In noise conditions, speech-spectrum noise (SSN) was played in synchrony with syllables. SSN was generated by spectrally modulating white noise using the long-term average spectrum of 5,000 Mandarin sentences produced by 10 young female speakers, followed by low-pass filtering at 5 kHz (Wu et al., 2005). For each participant, the intensity of SSN was adjusted individually to produce signal-to-noise ratios (SNRs) at a moderate level in a pretest before the experimental session.

### TACS Stimuli

Two rubber electrodes (5 × 7 cm; 35 cm^2^), covered by sponge pads and wetted with a 0.9% saline solution (5 mL per electrode), were placed on the T7 and T8 locations according to the International 10–20 EEG system after localization with an EEG cap (Figure 2D–E). This montage was designed to target bilateral auditory cortical regions, since speech perception recruits auditory areas in both hemispheres (Hullett et al., 2016), although some speech-related processes may show hemispheric asymmetries (Liang & Du, 2018; Tang et al., 2021). Similar bilateral stimulation protocols have been used in previous auditory tACS studies (e.g., Keshavarzi et al., 2020; Zoefel et al., 2018). The electrodes were connected to a neurostimulation device (Soterix Medical 1 × 1 Transcranial Electrical Stimulation Model 2001 Low-Intensity Stimulator). The resistance between the electrodes of each device was set to be no more than 10 kΩ.

Stimulation was delivered continuously for about 20 min. For each participant, stimulation intensity was individually adjusted to remain below the perception threshold, ensuring that participants did not reliably detect the presence of electric stimulation. Following procedures commonly used in previous tACS studies (Rufener, Zaehle, et al., 2016), threshold estimation was conducted using a staircase procedure. Two sets of 5 s sinusoidal testing currents at 4 Hz and 40 Hz were applied, respectively. The stimulation amplitude started at 0.8 mA and was increased in steps of 0.1 mA up to a maximum of 1.5 mA. The procedure was stopped once participants reported a noticeable skin sensation, and this value was defined as the individual perception threshold. The stimulation intensity used in the main experiment was then set below this threshold and kept constant throughout the session. Across participants, the mean stimulation amplitude was 0.83 mA (*SD* = 0.14 mA) for theta tACS and 1.02 mA (*SD* = 0.17 mA) for gamma tACS.

### Experimental Procedure

All participants undertook four experimental sessions on four separate days. Each participant came four times to finish one pretest session and three formal experimental sessions, respectively. Participants were seated in a soundproof room. The pretest began with questionnaire surveys (see Questionnaires 1–4 of the Supplementary Materials), including an informed consent form, demographic surveys, music experience evaluations, and a handedness inventory. Following this, participants completed a pure-tone average test and an electric stimulation toleration test. Subsequent psychoacoustic tests first established individualized ambiguity ranges for tone and consonant continua in quiet conditions, followed by a syllable-in-noise identification task to determine individualized perceptual thresholds for these features in noisy environments. The experiment was implemented using custom-written stimulus presentation scripts in MATLAB R2016 (Liang et al., 2023b).

To estimate the individualized perceptual ambiguity ranges for pitch and voice onset time (VOT) continua, participants undertook tone or consonant identification tasks using a 9 × 9-step subset from a 54 × 54-step continuum matrix, applying the method of constant stimuli. During each trial, participants listened to a syllable and were required to identify the tone or consonant category to which it belonged, disregarding the other dimension. The boundaries of the perceptual ranges were manually identified by experimenters. Based on the individualized parameters, we synthesized one 5-step tone and one 5-step consonant continuum for each participant. Re-synthesized syllables were longer than a period of theta waves (mean = 0.378 s > 0.250 s, *SD* = 0.005 s), but a theta window covers enough time samples for consonant or tone identification, so we would not scale the syllables to sacrifice authenticity. Nevertheless, differences in VOT length between step 1 and step 3 syllables surrounding the PSE were similar to a gamma period (mean = 0.020 s ≈ 0.025 s, *SD* = 0.005 s), matching the target band modulated by gamma tACS.

Individualized SNR levels were also estimated via the constant stimuli method, where participants performed tone and consonant identification tasks utilizing clear syllables ([ti^55^] and [ti^35^] for tones, [ti^55^] and [t^h^i^55^] for consonants), obscured by SSN. The SSN was presented in synchronization with the syllables with 10-ms linear rise and decay envelopes. The individualized SNR levels were defined as the point where participants reached approximately 85% accuracy, ranging from −16 dB to 2 dB for tone tasks and −12 dB to 12 dB for consonant tasks.

The following three formal experimental sessions were theta band (4 Hz) tACS, gamma band (40 Hz) tACS, and a sham session (Figure 2A–C). The order of formal sessions was counterbalanced across participants using a balanced Latin square procedure, with a between-session gap of at least 5 days. Online tACS was applied during the experiment in each session. In sham conditions, 30-second short currents were only applied during the ramp-up and ramp-down periods before and after the experiment. Each session consisted of four blocks: tone/consonant identification in either a quiet or noisy environment. Block order was counterbalanced across participants and sessions using a balanced Latin square design. One block included four mini-blocks, with each mini-block randomly presenting all syllables in the continuum. To improve the reliability of slope fits, extra trials near the PSE were included in each mini-block, resulting in a total of 156 trials (39 trials per mini-block). During each trial, participants listened to a syllable and made a speeded perceptual decision via pressing the “<” or “>” keyboard key with their left or right index or middle fingers to judge the tone (tone1 or tone2) or consonant (“d” or “t”) of the heard syllable. Participants’ responses and RTs were recorded. The responding hand and key assignments were counterbalanced across participants. A maximum 5-s resting period was allowed after sound onset for participants to respond. The next trial began automatically 0.5 s (range: 0.4–0.6 s) after the previous response or the trial’s end.

Before each formal session, participants completed practice tasks to familiarize themselves with the experimental procedure. The practice task before the first session involved brief tone/consonant identification tasks using a different continuum matrix ([p]–[p^h^] × tone1–tone2 in Mandarin), whereas the practice tasks before the second and third sessions included a short version of the formal experiment. At the end of each electric stimulation, participants rated the intensity of the perceived sensation on a 4-point scale (see Questionnaire 4 of the Supplementary Materials), with 1 and 4 indicating no sensations and strong sensations, respectively (no significant differences were found among three tACS conditions: *F*(2, 54) = 1.87, *p* = 0.16, *η*^2^ = 0.065; Gamma: mean = 16.26, *SD* = 3.62; Theta: mean = 15.42, *SD* = 3.75; Sham: mean = 14.31, *SD* = 3.61).

### Statistical Analysis

#### Fitting psychometric functions

First, we applied binary coding to participants’ responses (i.e., 0 for “d”/“tone1” and 1 for “t”/“tone2”) in the tone and consonant identification tasks and fitted a four-parameter logistic model using the nonlinear *lsqcurvefit* function in MATLAB R2016a, with morphing steps in a continuum as the explanatory variable and responses as the response variable. The slope of the fitted curve at the mid-point served as an index of perceptual sensitivity (Kingdom & Prins, 2016). The formula of this curve is shown below:

$$\hat{Y}=\frac{a-b}{1+e^{-\sigma \left(X-\mu \right)}}+b$$ (1)

Here, $\hat{Y}$ is the estimated psychometric function (identification curve). *X* is the steps of a continuum. *a* and *b* are the upper and lower asymptotes of the curve. *μ* is the PSE. *σ* is the slope of the curve at the PSE. Slopes of psychometric functions served as the response variable. The lower the slopes, the weaker the participant’s capacity to categorize phonemes. A four-parameter logistic function was employed instead of a simplified model with asymptotes fixed at 0 and 1. This decision was motivated by two main considerations. First, it was unrealistic to assume perfect performance (i.e., 100% accuracy) even when the tone or consonant was highly discriminable, particularly under noise-masking conditions. Second, to mitigate overfitting, the upper and lower asymptotes for each condition were determined by an unconstrained fit to the average data and were subsequently fixed for each block. For each condition, slopes outliers exceeding three standard deviations (*SD*) from the mean were excluded. However, given that tACS may exert inhibitory effects and potentially induce fitting failure, we adopted a correction procedure for participants with a valid slope in sham but an outlier slope in tACS conditions. Specifically, the outlier slope was replaced with a floor value derived from the corresponding sham blocks (Liang et al., 2023a). This floor value was computed as: F = exp(mean(log(D)) − 2 * *SD*(log(D))), where D was the slope estimates in sham conditions. We adopted this step to minimize bias introduced by outliers while preserving potential tACS effects. Subsequently, both sham and adjusted tACS slope values were then log-transformed to correct for skewness. To assess the statistical significance of tACS effects for each task (tone/consonant perception under quiet/noise conditions), we conducted a permutation test. Participants’ slope values were shuffled between tACS and sham blocks. For each iteration, the mean difference between conditions was calculated. This procedure was repeated 10^5^ times to generate a null distribution of mean differences. The *p* value was determined by ranking the observed effect within the permutation distribution, using the following formula: *p* = (ranking + 1) / (10^5^ + 1).

#### Linear mixed-effects modeling of RTs

We then tested whether tACS significantly altered RTs in each condition by using linear mixed-effects (LME) models. To address the first question and test three associated hypotheses, we established a global model (Model 1) to comprehensively examine the effects of tACS on RTs under different task types and environments (quiet or noisy). The fixed effects in the model included *Stimulus type* (theta vs. gamma tACS), *Task type* (tone vs. consonant identification), and *Environment* (quiet vs. noisy). The *Participant* was included as a random intercept. To focus on testing for tACS effects and to avoid model convergence problems, no random effects were modelled for slope in this study. The global model for RTs is specified as follows:

RTs ∼ Stimulus type * Task type * Environment + (1|Participant)  (Model 1)

Given that tone and consonant are two distinct speech cues that may rely on different processing mechanisms (i.e., involving different neural processing mechanisms) (Meyer, 2018; Rufener, Zaehle, et al., 2016), it is worthy examining the effects of tACS separately for the two *Task types* (tone vs. consonant identification). Therefore, two sub-models (Model 2) for tone and consonant tasks were constructed to investigate further the interactions between tACS and environment, respectively. Model 2 removed the *Task type* factor from the original model (Model 1). In these models, *Stimulus types* and *Environment* were included as fixed effects, with the *Participant* modeled as a random intercept. The best models for RTs are specified as follows:

RTs ∼ Stimulus type * Environment + (1|Participant)  (Model 2 Tone)

RTs ∼ Stimulus type * Environment + (1|Participant)  (Model 2 Consonant)

All models were implemented using the lme4 package in RStudio 1.4.1103 (R version 4.0.4), and model tests were conducted using the lmeTest package. *P* values were corrected for multiple comparisons using the false discovery rate (FDR) method across all tACS conditions within each task.

#### Hierarchical drift-diffusion modeling

To gain deeper insight into the cognitive mechanisms through which tACS may influence Mandarin syllable perception, particularly in terms of how tACS modulates decision-related processes beyond surface-level behavioral measures, we further applied a hierarchical drift-diffusion model (HDDM; Wiecki et al., 2013) to jointly model trial-level categorical responses and RTs across participants. This model describes perceptual decision making as an evidence accumulation process modulated by four key parameters: *a* (boundary threshold), *v* (drift rate), *z* (starting point), and *t* (non-decision time). The analyses were conducted using the HDDM package (version 0.9.8) based on Python 2.7.

HDDM analyses focused on the quiet condition, as only this condition showed concurrent modulation of categorical responses and RTs, allowing meaningful interpretation of latent decision parameters. In contrast, in the noise condition, tACS effects were limited to RTs, making HDDM decomposition less informative.

To test whether tACS significantly affected the parameters *a*, *v*, and *z*, we constructed two linear regression models for each task (tone vs. consonant identification), separately assessing the effects of gamma and theta stimulation. *Stimulus type* was dummy-coded with the sham condition serving as baseline. The probability that the parameter posterior distributions deviated from zero (two-tailed test) reflected the confidence (the *p* value) in rejecting the null hypothesis (i.e., no effects of tACS). All *p* values were then FDR-corrected for multiple comparisons across all tACS conditions. Although non-decision time (*t*) was included in the model, it was treated as an intercept-only parameter to avoid overfitting and prevent overinterpretation of its psychological significance (Weindel et al., 2021).

## RESULTS

### Theta tACS Impairs Consonant Sensitivity in Quiet

**Figure 3.**
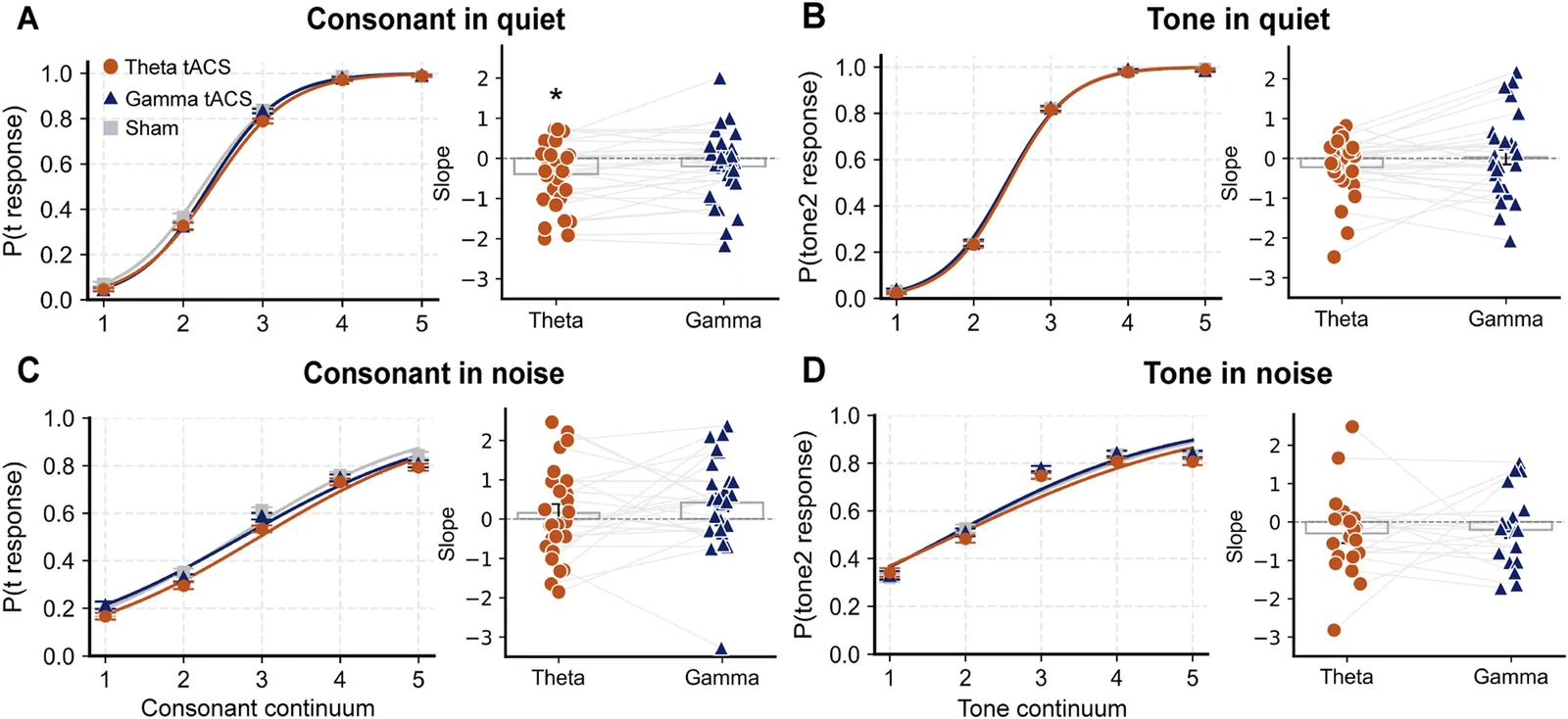
Sigmoid psychometric functions and corresponding slope estimates at the points of subjective equity (PSE, step3) across five-step continua in different transcranial alternating current stimulation (tACS) and listening conditions. (A–D) Each panel contains two plots: the left plot shows the psychometric function (the proportion of categorical responses across continuum steps), and the right plot shows the corresponding PSE slope for each stimulation condition. (A, B) In quiet conditions, the proportions of “t” responses (A, consonant continuum) or “tone 2” responses (B, tone continuum) were plotted along continuum steps. (C, D) The same measures were shown under background noise. Lines in the left sub-plots of each panel indicate mean responses across participants for each of the three stimulation conditions (theta tACS: orange; gamma tACS: blue; sham: gray), with error bars representing standard error. All psychometric functions reflect increased “t” or “tone 2” responses from acoustic step 1 to 5. Noted that, in A, theta tACS elicited a shallower slope compared to gamma tACS and sham, suggesting a reduced sharpness of the category boundary under theta stimulation. Compared with sham, only theta tACS lowered the psychometric slope in the consonant task in quiet (*p_*fdr*_ = 0.031, two-tailed paired *t*-test). No observable differences in slope were observed between stimulation conditions in tone perception (B, D) or in the consonant in noise condition (C). In the right subplots of each panel, each dot represents one participant; gray lines connect paired data points across stimulation conditions. Bars indicate group means.

To assess how tACS influenced Mandarin syllable perception, we first analyzed psychometric functions across all experimental conditions (Figure 3). The slope of each function served as an index of perceptual sensitivity for consonant and tone categorization (Figure 3). Critically, for consonant perception in the quiet condition (Figure 3A), theta tACS significantly lowered the psychometric slope (compared with sham: *p*_*fdr*_ = 0.031, Cohen’s *d* = −0.499), indicating a decrease in perceptual sensitivity; in contrast, gamma tACS did not significantly affect the slope (compared with sham: *p*_*fdr*_ = 0.216, Cohen’s *d* = −0.231), and only marginal significant difference was found between theta and gamma tACS conditions (gamma > theta: *p*_*fdr*_ = 0.083, Cohen’s *d* = 0.36). However, neither theta nor gamma tACS affected slope values for tone perception in quiet (all *p* > 0.05, Figure 3B). When noise was added, tACS had no significant effects on psychometric slopes in either tone or consonant perception (all *p* > 0.05, Figure 3C–D).

We next examined whether tACS altered response bias by analyzing the PSE in consonant categorization (see Supplementary Materials Figure S1). In the quiet conditions, both gamma and theta tACS induced marginal shifts in PSE relative to sham (gamma: *p*_*fdr*_ = 0.081, Cohen’s *d* = 0.298; theta *p*_*fdr*_ = 0.081, Cohen’s *d* = 0.325), hinting at a potential, albeit weak, modulation of categorical boundaries. A similar trend was observed for theta tACS in noise (compared with sham: *p*_*fdr*_ = 0.083, Cohen’s *d* = 0.430), suggesting that theta stimulation might subtly bias perceptual thresholds even in challenging listening environments. However, no significant PSE effects were found for tone perception in either quiet or noise conditions (all *p* > 0.05).

In summary, the psychometric effects of tACS were mainly observed in consonant perception under quiet conditions: theta tACS significantly reduced perceptual sensitivity (slope decrease, Figure 3A), suggesting impaired encoding of phonemic contrasts. Marginal shifts in PSE were also observed for both theta and gamma tACS, but only in consonant categorization. These findings indicated that theta-band neural activity is causally involved in phoneme boundary perception in Mandarin. Such effects were primarily observed in consonant categorization under quiet listening conditions.

### Theta tACS Prolonged RTs Across Tasks and Environments but Gamma Only Delays Tone Judgment in Quiet

We then proceeded to comprehensively characterize the temporal dynamics of tACS effects using LME models (Table 1). This approach allowed us to account for both fixed effects of experimental conditions and random variation across participants while examining RT differences. The results are reported in an analysis of variance (ANOVA) format.

**Table 1.** Modulations of transcranial alternating current stimulation (tACS) effects on RTs in different conditions (seconds, *Mean* ± *SD*).

**Stimulus type**	**In quiet**		**In noise**	
	**Consonant**	**Tone**	**Consonant**	**Tone**
Theta tACS	0.77 ± 0.24	0.81 ± 0.23	0.88 ± 0.27	0.96 ± 0.33
Gamma tACS	0.76 ± 0.22	0.80 ± 0.23	0.86 ± 0.27	0.94 ± 0.33
Sham	0.75 ± 0.24	0.79 ± 0.24	0.86 ± 0.27	0.95 ± 0.36

In Model 1, a significant main effect of *Stimulus type* was observed (*χ*^2^(2) = 54.480, *p* < 0.001). Post hoc tests demonstrated that theta tACS significantly prolonged RTs relative to both sham (*β* = −0.017, *SE* = 0.002, *t* = −6.998, *p* < 0.001) and gamma tACS (*β* = −0.013, *SE* = 0.002, *t* = −5.522, *p* < 0.001), whereas gamma tACS did not differ significantly from sham (*β* = −0.003, *SE* = 0.002, *t* = −1.479, *p* = 0.139). A significant main effect of *Task type* emerged (*χ*^2^(1) = 893.890, *p* < 0.001), with longer RTs observed for tone compared to consonant categorization (*β* = −0.059, *SE* = 0.002, *t* = –29.915, *p* < 0.001). Additionally, RTs were strongly modulated by *Environment* (*χ*^2^(1) = 4389.810, *p* < 0.001), with longer RTs observed in noise versus quiet conditions (*β* = −0.130, *SE* = 0.002, *t* = –66.262, *p* < 0.001).

A significant interaction was found between *Stimulus type* and *Environment* (*χ*^2^(2) = 6.410, *p* = 0.041), indicating that tACS effects were modulated by environment. Simple effects analysis revealed that, in quiet environment, RTs under both theta tACS (*β* = −0.021, *SE* = 0.003, *t* = −6.230, *p* < 0.001) and gamma tACS (*β* = −0.009, *SE* = 0.003, *t* = −2.772, *p* = 0.006) were significantly longer compared to sham, with theta tACS exerting an even stronger effect than gamma (*β* = −0.012, *SE* = 0.003, *t* = −3.458, *p* < 0.001). In noisy environment, theta tACS kept lengthening RTs relative to sham (*β* = −0.013, *SE* = 0.003, *t* = −3.669, *p* < 0.001) and gamma tACS (*β* = −0.015, *SE* = 0.003, *t* = −4.351, *p* < 0.001), whereas no significant difference was observed between gamma tACS and sham (*β* = 0.002, *SE* = 0.003, *t* = 0.679, *p* = 0.497). This pattern suggested that theta tACS consistently impeded response speed, regardless of environmental noise, while gamma tACS showed weaker and condition-specific effects, which were constrained to the unchallenging listening (quiet) environment.

An additional significant interaction was observed between *Task type* and *Environment* (*χ*^2^(1) = 118.90, *p* < 0.001). Simple effects analysis indicated that RTs in the tone identification task were significantly longer than those in the consonant identification task, both in quiet (*β* = −0.0374, *SE* = 0.00278, *t* = –13.452, *p* < 0.001) and in noise (*β* = −0.0804, *SE* = 0.00279, *t* = –28.843, *p* < 0.001). All other interactions were not statistically significant (all *p* > 0.05).

To examine whether tACS effects were modulated differentially by *Task types*, we analyzed consonant and tone identification tasks separately using the same LME model (RTs ∼ Stimulus type * Environment + (1|Participant)).

**Figure 4.**
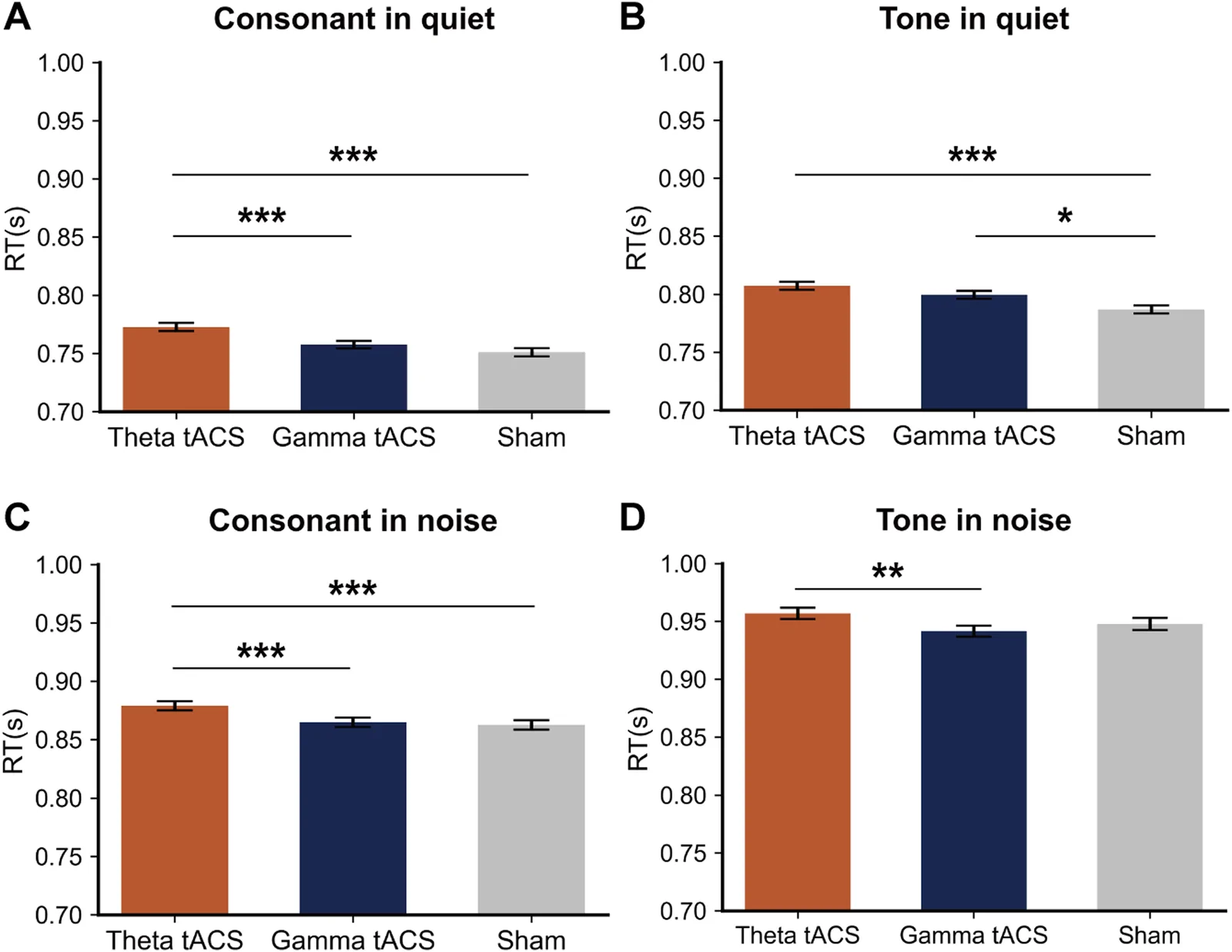
Reaction times (RTs) across transcranial alternating current stimulation (tACS) conditions in consonant and tone identification tasks in quiet and noise. (A) In the consonant tasks in quiet, theta tACS resulted in significantly longer RTs compared to both gamma tACS and sham. (B) In the tone tasks in quiet, both stimulation conditions led to longer RTs than sham. (C) In the consonant tasks in noise, RTs were significantly prolonged under theta tACS compared to both gamma tACS and sham. (D) In the tone tasks in noise, only theta tACS led to significantly longer RTs than gamma tACS. *p_*fdr*_ < 0.05. **p_*fdr*_ < 0.01. ***p_*fdr*_ < 0.001. Bars indicate group means; error bars indicate standard error.

In Model 2 Consonant (Figure 4A, C), significant main effects of *Stimulus type* (*χ*^2^(2) = 43.641, *p* < 0.001) and *Environment* (*χ*^2^(1) = 1923.536, *p* < 0.001) were observed. Post hoc tests showed that theta tACS again slowed RTs versus sham (*β* = −0.019, *SE* = 0.003, *t* = −6.278, *p* < 0.001) and gamma tACS (*β* = −0.015, *SE* = 0.003, *t* = −4.915, *p* < 0.001), while gamma tACS and sham did not differ (*β* = −0.004, *SE* = 0.003, *t* = −1.366, *p* = 0.172). The interaction between *Stimulus type* and *Environment* was non-significant (*χ*^2^(2) = 1.015, *p* = 0.602).

In Model 2 Tone (Figure 4B, D), analysis of RTs revealed a significant main effect of *Stimulus type* (*χ*^2^(2) = 17.933, *p* < 0.001). Post hoc tests indicated that theta tACS prolonged RTs relative to sham (*β* = −0.015, *SE* = 0.004, *t* = −4.007, *p* < 0.001) and gamma tACS (*β* = −0.012, *SE* = 0.004, *t* = −3.186, *p* = 0.002), while gamma tACS effects did not differ from sham (*β* = −0.003, *SE* = 0.004, *t* = −0.822, *p* = 0.411). A significant main effect of *Environment* was also observed (*χ*^2^(1) = 2582.679, *p* < 0.001), with RTs being significantly longer in noise conditions compared to quiet conditions (*β* = −0.152, *SE* = 0.003, *t* = –50.820, *p* < 0.001).

The interaction between *Stimulus type* and *Environment* was significant (*χ*^2^(2) = 6.664, *p* = 0.036). Simple effects analyses in Figure 4B showed that, in quiet, both theta (*β* = −0.020, *SE* = 0.005, *t* = −3.950, *p* < 0.001) and gamma tACS (*β* = −0.012, *SE* = 0.005, *t* = −2.392, *p* = 0.025) significantly increased RTs compared to sham, with no significant difference between theta and gamma tACS (*β* = −0.008, *SE* = 0.005, *t* = −1.558, *p* = 0.119). In noise (Figure 4D), neither theta nor gamma tACS significantly differed from sham (all *p* > 0.05). However, RTs under theta tACS were significantly longer than those under gamma tACS (*β* = −0.015, *SE* = 0.005, *t* = −2.946, *p* < 0.01). Notably, the effect of gamma tACS was only present in the quiet condition.

In sum, these results from both models indicated that theta tACS reliably resulted in longer RTs compared to sham across tasks and environments, suggesting a broad impact on processing efficiency. By contrast, gamma tACS showed more selective effects, increasing RTs mainly during tone perception in quiet. This extends our findings on the psychometric slope by providing evidence for a role of theta activity in temporal integration mechanisms and of gamma activity in tone-related auditory processing during Mandarin syllable perception.

### Theta and Gamma tACS Modulated the Perceptual Decision Making in Mandarin Syllables

**Figure 5.**
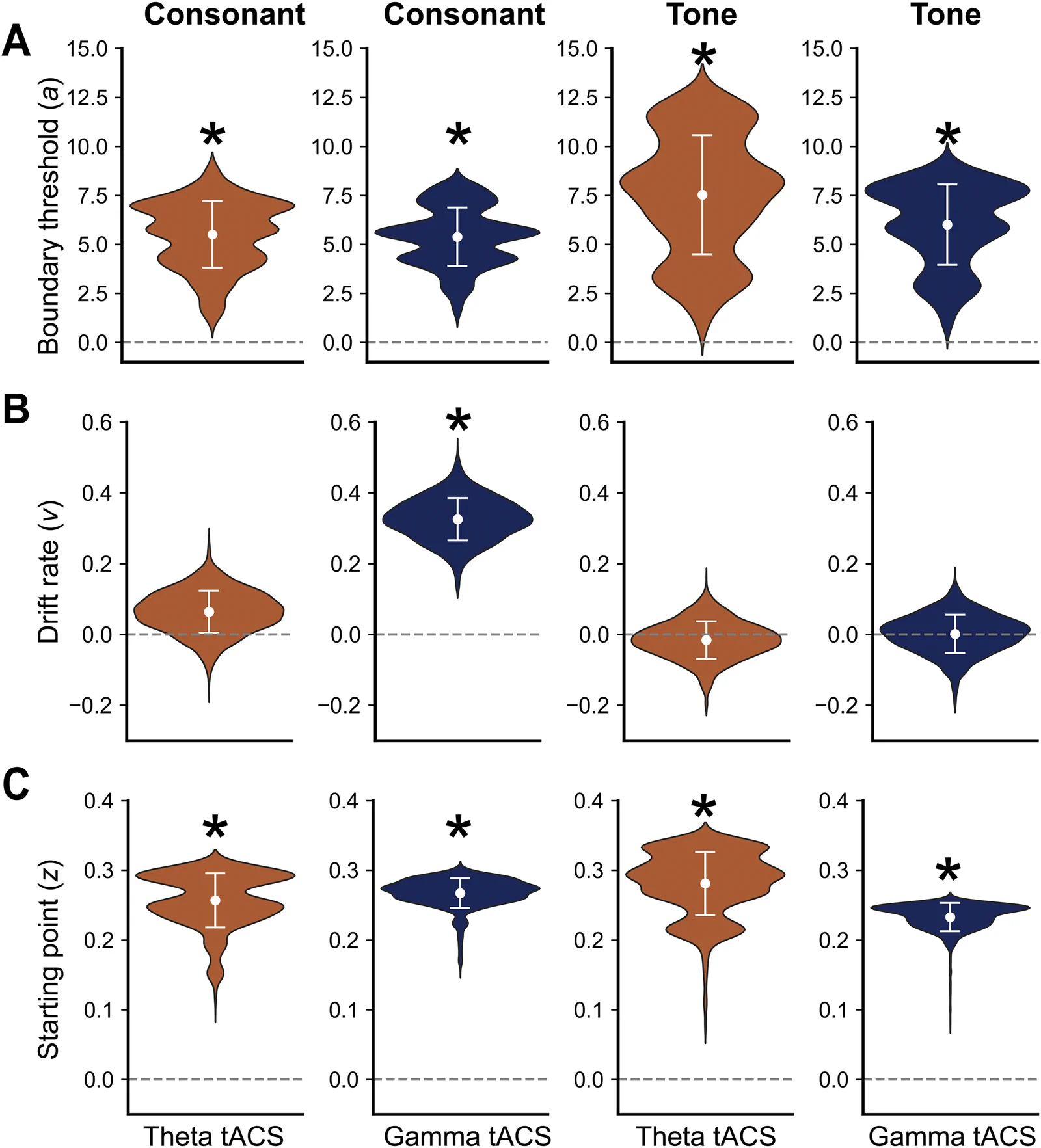
TACS effects on HDDM parameters in the quiet condition across tasks. Each row displays violin plots of one parameter estimated from hierarchical Bayesian modeling: boundary threshold (*a*, A) drift rate (*v*, B), and starting point (*z*, C). Within each sub-plot, data were plotted separately for consonant (left) and tone (right) identification tasks under theta (orange) and gamma (blue) transcranial alternating current stimulation (tACS). Error bars represent *SEM*. *p_*fdr*_ < 0.05 (two-tailed). (A) Boundary threshold (*a*), indicating the amount of evidence required to trigger a decision, was significantly increased in all conditions. Larger values reflect more cautious decision making. (B) Drift rate (*v*), reflecting the efficiency of evidence accumulation, showed a significant rise only in the consonant task under gamma tACS. Larger values indicate more efficient stimulus-driven processing. (C) Starting point (*z*), representing prior bias toward a particular response, significantly deviated from zero in all conditions. Compared with Sham, positive values indicate a prior bias toward “d” or tone 1; negative values indicate a bias toward “t” or tone 2.

To dissect which cognitive subprocesses of speech perceptual decision making are supported by theta and gamma neural activity, we applied HDDM to jointly model categorical responses and RTs. This approach decomposes decision making into three interpretable parameters: boundary threshold (*a*) reflecting threshold for evidence accumulation, drift rate (*v*) indexing evidence accumulation rate, and starting point (*z*) capturing initial response bias. TACS effects on these parameters were quantified using linear regression models comparing each stimulation condition with sham (Figure 5).

In the consonant identification task, gamma tACS significantly elevated the boundary threshold (*a*), indicating that participants required more evidence to reach a decision. It also enhanced the drift rate (*v*), reflecting a faster accumulation of sensory information. Additionally, gamma stimulation shifted the starting point (*z*) toward the “d” response, suggesting a stronger initial bias toward that choice (all *p*_*fdr*_ < 0.001, two-tailed test). Theta tACS similarly raised boundary threshold (*a*) and altered starting point (*z*) (all *p*_*fdr*_ < 0.001, two-tailed test), but did not significantly alter the drift rate (*v*) (*p*_*fdr*_ = 0.144).

In the tone identification task, both gamma and theta tACS significantly increased the parameter *a*, with all posterior distributions deviating from zero (all *p*_*fdr*_ < 0.001, two-tailed test). This indicated that participants required more evidence to make a decision after gamma or theta activity was perturbed. TACS also significantly perturbed the parameter *z*, suggesting a response bias shift toward tone 1 choice. However, neither gamma (*p*_*fdr*_ = 0.463) nor theta tACS (*p*_*fdr*_ = 0.376) significantly affected the drift rate (*v*), suggesting that the speed of evidence accumulation remained unchanged.

To summarize, theta and gamma tACS consistently increased the boundary threshold (*a*) and biased the starting point (*z*) upward across tasks, suggesting increased caution and altered decision biases. Notably, only gamma tACS led to a significant increase in drift rate (*v*) during consonant perception, indicating a unique increase in evidence accumulation speed.

## DISCUSSION

In this study, we applied theta- and gamma-band tACS to the bilateral temporal cortex of native Mandarin speakers while they performed Mandarin syllable (consonant and tone) identification tasks. We examined how stimulation in these two frequency bands affected task performance, to test whether neural activity in these bands causally supports Mandarin syllable perception. Furthermore, we assessed whether these mechanisms are modulated by the environment (quiet vs. noisy environment).

### Shared Neural Mechanisms for Mandarin Consonant and Lexical Tone Perception Are Supported by Theta Activity

The present study provides causal evidence that theta neural activity plays a key role in consonant and lexical tone perception in Mandarin. Moreover, theta tACS not only slowed perceptual decisions but also significantly reduced the psychometric slope for consonant identification in quiet conditions, indicating less distinct category boundaries and impaired perceptual sensitivity. In contrast, gamma activity exerted more selective influences, particularly in lexical tone perception under quiet conditions, and did not significantly alter psychometric slopes. While psychometric slope indexes perceptual sensitivity, RT reflects processing efficiency and the cognitive resources involved in perceptual decision making (Li et al., 2024; Peters et al., 2024). In our results, theta tACS reliably increased RTs across tasks while selectively reducing the slope for consonant perception, suggesting that the stimulation may impose additional processing demands rather than degrading perceptual representations. The lack of significant PSE shifts further suggests that theta stimulation did not systematically bias participants toward one response category or another, but rather reduced the sharpness of phonological category boundaries. Taken together, the combined pattern of RT and slope results largely supports Hypothesis 3, which states that consonants and lexical tones in Mandarin are identified as holistic syllabic units that encapsulate both suprasegmental and segmental cues, subserved by theta activity (Figure 1C).

Theta activity has been found to track speech rhythm and integrate slowly varying suprasegmental features in speech (Meyer, 2018; Teoh et al., 2019). Consistent with it, the present findings suggest that theta activity supports syllable-level speech processing, particularly for consonants embedded within Mandarin syllables. Reduced psychometric slopes under theta stimulation indicate less distinct phonological category boundaries. This syllable-level role may be particularly relevant in Mandarin, where lexical tones are tightly integrated with syllable structure (He et al., 2023; Ni et al., 2023). Our results suggest that the functions of theta neural activity are not limited to supporting suprasegmental tone integration but extend broadly to segmental phonemes (e.g., consonants).

One explanation is that consonants and lexical tones in Mandarin may share integrated syllabic representations rather than being perceived as independent linguistic units (Yu et al., 2022). Indeed, in current Mandarin, lexical tones function as phonetic features to determine meanings, resembling consonants (Huang & Liao, 2017; Liang & Du, 2018). Moreover, one recent behavioral study using consonant/tone identification tasks with the same set of syllables also supported this hypothesis (Liang et al., 2023a). Instead of using separate consonant and lexical tone continua, however, Liang et al. (2023a) used one consonant-tone continua matrix for both tasks, with each synthesized syllable having both VOT and F0 contour modulated. Results showed that when the lexical tones or consonants were half-ambiguous (i.e., step 2 or 4) for certain syllables, the slopes of the psychometric curves in the unrelated identification tasks (e.g., consonant identification for syllables with half-ambiguous tones, or vice versa) were significantly lowered compared with the other conditions (see, e.g., Supplementary Figure 3 in Liang et al., 2023a). This pattern closely resembles the present findings, in which theta stimulation selectively reduced consonant slopes without shifting PSEs, suggesting that theta tACS did not bias participants toward a specific category but less distinctive consonant boundaries within an integrated syllable representation. Moreover, evidence from ERP study (Zhao et al., 2011; Zou et al., 2020) and phonetic research (Kang & Xu, 2024; Liu & Xu, 2023) has shown a high degree of synchronization between tone and vowel (another type of phoneme) processing, supporting the holistic view of consonant and tone perception.

This finding differs from previous tACS studies on English syllable identification, which have found that stimulation in the gamma band selectively modulates the perception of phonemes (Marchesotti et al., 2020; Rufener, Oechslin, et al., 2016; Rufener, Zaehle, et al., 2016), corresponding to their temporal modulation rates of around 40 Hz. This difference may stem from both linguistic structure and the methods of stimulation employed. Firstly, in a tonal language such as Mandarin, phonetic perception may rely heavily on the integration at the syllable level, with consonants embedded within temporally extended syllabic and tonal contexts. In contrast, phoneme perception in non-tonal languages relies more on the phoneme level. Secondly, the effect of different stimulation timing can be another factor. In previous studies, tACS was temporally aligned with stimulus or endogenous neural oscillatory activity (e.g., Keshavarzi et al., 2020; Zoefel et al., 2018), which may facilitate neural entrainment and support phonemic processing. In the present study, stimulation was temporally jittered rather than phase-locked, which may have reduced phase entrainment of neural oscillations, thereby impairing the processing of consonantal cues.

However, the absence of significant theta-related slope changes in lexical tone perception suggests that consonants and tones may not be equally susceptible to theta-band disruption. One possible explanation is that tonal categories in Mandarin may be more robust and perceptually salient than consonant categories in the present task (Fu et al., 1995; Liang et al., 2023a). Lexical tones unfold over longer temporal intervals and are supported by multiple redundant acoustic cues, including F0 contour, pitch height, and duration. By contrast, consonant identification often depends on brief and rapidly changing acoustic cues, making consonant category boundaries more susceptible to disruption of theta-mediated syllable integration.

An alternative explanation is that the experiment itself used meaningful words that could trigger holistic semantic processes. However, in our experiments, participants were not explicitly informed of the Chinese characters or meanings of the syllables, and it was difficult for them to infer the meanings because a single Chinese syllable often corresponds to multiple characters and meanings. Moreover, Liang et al. (2023a) used the same experimental tasks and materials to selectively modulate the perception of lexical tone and consonants with transcranial magnetic stimulation, suggesting that the current paradigm can effectively elicit sub-syllabic representations.

Importantly, although theta tACS effects on psychophysical sensitivity were attenuated or even absent under noisy conditions (Figure 3A, C), its inhibitory influence on RTs in both consonant and tone tasks persisted (Figure 4). This pattern suggests that adverse listening conditions may shift the dominant processing mechanisms engaged during syllable perception. Specifically, increased perceptual difficulty under noise may induce changes in listening strategy and attentional allocation, leading to greater reliance on top-down compensatory processes, such as sensorimotor integration and verbal working memory (Alain et al., 2018). Such compensatory mechanisms are thought to be supported more strongly by oscillatory activity in higher frequency bands (e.g., mu rhythm in alpha and beta range; Jenson et al., 2014; Saltuklaroglu et al., 2018), rather than by auditory-driven theta-band temporal sampling. As a result, the reduced or absent effect of theta tACS on perceptual sensitivity under noisy conditions may reflect a decreased reliance on theta-mediated auditory processing, rather than a complete loss of theta-related function.

At the same time, the persistence of RT effects suggests that theta stimulation may continue to influence overall processing efficiency or perceptual load, even when sensitivity-related measures are less affected. This pattern suggests that syllable perception under adverse conditions may involve flexible reconfiguration of processing strategies.

Based on this, lexical tones should be understood within the overall context of the language, recognizing their synchronic and diachronic relationships with other linguistic elements (e.g., phonemes, syllables), rather than treating them as isolated acoustic features. For example, from a historical linguistics perspective, the [ti^55^], [ti^35^], [t^h^i^55^], and [t^h^i^35^] used in our experiment could have originated from four syllables with only consonant contrasts (e.g., additional voiced vs. voiceless contrasts; Baxter & Sagart, 2014; Michaud & Sands, 2020; Norman, 1988). Consistent with our results, this evolutionary trajectory implies that Mandarin consonants and lexical tones are processed at an integrated syllable level, as segmental consonant contrasts can be transformed into suprasegmental lexical tone contrasts. Additionally, other studies suggest that the formation of the current Chinese tonal system is linked to the disyllabification of the language (Shi, 2023), indicating that the cognitive processing of tones may be closely associated not only with phonemes but also with higher level prosodic units such as syllables. This aligns with a morphophonological perspective, wherein suprasegmental features, such as tones, are mapped onto and interact with the syllabic and morphological structure of the language, rather than being processed in isolation.

### Theta and Gamma Activity Support the Perceptual Decision in Mandarin Syllables

Results from the HDDM revealed that theta tACS systematically increased the decision boundary (*a*, Figure 5A) and modified the starting point (*z*, Figure 5C). Besides hampering the auditory sampling and impairing speech perception (Meyer, 2018), indicated by the increased amount of perceptual evidence required for decision (*a*), theta tACS also affected the response bias (*z*) that determines the decision relying more on memory instead of the upcoming speech input. Meanwhile, since we applied random inter-trial intervals, it is unlikely that this response bias was caused by tACS-induced phase resetting of neural oscillations (Riecke et al., 2015), which would have lead to a systematic change in the sampling of VOT and pitch contour. Therefore, the modulation of response bias by theta tACS may reflect the role of theta activity in supporting memory functions or general motor decision-making processes; however, future studies are needed to draw a solid conclusion.

More importantly, gamma tACS also exerted similar effects on the *a* and *z* as theta tACS did, while no significant gamma tACS effects on the psychometric slopes or RTs (except the RTs in lexical tone identification in quiet) were found. This indicates that gamma activity is also recruited in Mandarin syllable processing, but the recruitment tends to be compensatory rather than necessary. Indeed, gamma activity is critically involved in processing local, rapidly changing speech features (Fukuda et al., 2010; Lizarazu et al., 2019). Specifically, under quiet conditions, gamma activity may retain its advantage in processing fine-grained acoustic features, particularly pitch cues (Yellamsetty & Bidelman, 2018), which are less susceptible to noise. This corresponds to our finding that gamma tACS prolonged lexical tone identification but only in quiet. Indeed, one recent EEG study shows that the perception of lexical tone depends on temporal fine structure, supporting our results (Ni et al., 2023).

On the other hand, gamma tACS significantly enhanced the drift rate (*v*) for consonant processing. Noted that, gamma tACS did not modulate the psychometric slopes or RTs in consonant identification, regardless of environment. Previous studies showed that changes in drift rate indicate that top-down predictive processes enhance the rate of evidence accumulation. Thus, it is plausible that gamma activity was in fact actively supporting consonant perception, and hence was impaired by the gamma tACS (also indicated by the altered *a* and *v*). However, compensation from the higher levels of representations (e.g., syllable level) may counteract the inhibited, gamma-based, temporally fine phoneme encoding. In brief, gamma tACS modulated the HDDM parameters of perceptual decisions such as theta tACS, but only affected the performance of tone perception in quiet. This indicates that gamma activity is also involved in Mandarin phoneme perception, supporting the encoding of fast-changing features with fine-grained spectrotemporal patterns. However, their role is modulatory rather than causal, as the theta-supported holistic syllable representation can easily complement the degraded fine representations induced by impaired gamma activity.

### Limitations of the Study

Several limitations could affect the interpretations derived from the current results. First, this study did not directly measure neural activity, especially the modulatory effects of tACS. Although previous studies have shown that theta and gamma tACS can modulate activity at their corresponding frequencies (Ghiani et al., 2021), and a link between neural perturbations and behavioral changes (Riecke et al., 2015; Rufener, Zaehle, et al., 2016), future studies combining tACS with EEG would be valuable to directly assess frequency-specific entrainment and its relationship to behavioral effects.

Second, the observed effects of theta tACS on RT may partly reflect modulation of domain-general attentional or cognitive control processes, given that theta oscillations have been widely implicated in attention and executive functions (Menon & D’Esposito, 2022). However, based on our protocol to stimulate the temporal areas, these effects are more likely to reflect the modulation of auditory speech processing. Future studies incorporating independent attentional assessments may help disentangle speech-specific and domain-general mechanisms.

Third, as auditory perception activates bilateral auditory cortical regions, we employed this montage to simultaneously target both hemispheres. However, it may not only affect local oscillatory activity within each hemisphere but also influence interhemispheric coupling between bilateral auditory cortices (e.g., Assaneo et al., 2019). Accordingly, the present design does not allow us to disentangle local oscillatory modulation from changes in interhemispheric coupling. Future work should compare unilateral and bilateral stimulation and use EEG measures, such as coherence and phase-locking, to assess interhemispheric connectivity. In addition, manipulating the phase relationship between hemispheres may help clarify whether the present effects primarily reflect local oscillatory modulation or changes in interhemispheric communication.

### Conclusion and Implications

Collectively, the theta tACS effects observed in this study support our third hypothesis, Hypothesis 3 pointing out that the auditory encodings of Mandarin lexical tones and consonants are embedded in holistic syllable representations, supported by theta activity that facilitates the temporal sampling of syllabic cues. As tACS has been postulated to reveal causal effects by directly modulating neural activity (Herrmann et al., 2016), our study further implicates the causal role of theta activity in the perception and identification of Mandarin consonants and lexical tones, regardless of environment. Furthermore, our findings indicate that gamma neural activity is also engaged, but with a modulatory role, in resolving fine-grained spectrotemporal acoustic patterns, which can be complemented by the theta-based holistic syllable representations. Therefore, we propose that theta neural activity majorly contributes to Mandarin consonant and lexical tone perception through holistic syllable processing, but also with gamma compensation.

The results of this study also have potential implications for language education and clinical practice. For Chinese language education, existing research suggests that for learners of Chinese as a Second Language, the difficulty of learning tones is not reflected in the pronunciation of the pitch contours themselves, but rather in associating these contours with syllables (Hao, 2012). Therefore, tonal training could be integrated into the syllabic context (Pelzl et al., 2019) and its statistical regularities (Tang et al., 2024), and this study provides neural evidence for a teaching approach that embeds tone learning within syllable contexts. In terms of clinical practice, studies have shown that Mandarin-speaking individuals with developmental dyslexia exhibit deficits in tone perception (Cheung et al., 2009; Zhang et al., 2012), while patients with aphasia present with impairments in both tone perception (Zhang et al., 2023) and tone production (Chen et al., 2023). Based on the conclusions of the present study, future rehabilitation research could focus on developing language-specific rhythmic training paradigms for Mandarin or neuromodulation techniques centered around theta activity (Cheung et al., 2025). While rhythmic training (e.g., stress-based tasks) has been explored in stress-timed languages as English (Huss et al., 2011; Thomson et al., 2013), its application to syllable-timed languages such as Mandarin remains understudied. Our findings on syllable-level integration suggest that Mandarin-specific rhythm training (e.g., targeting syllabic timing rather than stress) could offer a novel and potent approach to simultaneously improve tone, phoneme, and syllable processing in Mandarin-speaking patients.

## ACKNOWLEDGMENTS

We greatly appreciate Yadong Niu from Peking University for providing the pure-tone-average testing scripts. Also, we thank Xuliang Zhang and Yixuan Feng from South China Normal University for their help with equipment set-up and data collection. We would like to thank Jiajie Zou from Ernst Strüngmann Institute for Neuroscience, and Yijun Fang from Faculty of Psychology and Educational Sciences, KU Leuven, for their suggestions on the manuscript, especially J. Z. for their assistance regarding auditory neural mechanisms, and Y. F. for their comments on historical linguistics and the potential significance of this research for improving language disorders.

## FUNDING INFORMATION

Ruiming Wang, National Natural Science Foundation of China, Award ID: 32371114. Keke Yu, Youth Fund Project of Ministry of Education of China on Humanities and Social Sciences Researches, Award ID: 22YJC190027. Ruiming Wang, Research Center for Brain Cognition and Human Development, Guangdong, China, Award ID: 2024B0303390003. Ruiming Wang, Striving for the First-Class, Improving Weak Links and Highlighting Features (SIH) Key Discipline for Psychology in South China Normal University.

## AUTHOR CONTRIBUTIONS

**Yaxuan Wang**: Formal analysis: Lead; Investigation: Lead; Methodology: Lead; Visualization: Lead; Writing – original draft: Lead. **Keke Yu**: Funding acquisition: Lead; Investigation: Supporting; Supervision: Equal; Writing – review & editing: Equal. **Shuqi Yin**: Investigation: Supporting; Methodology: Supporting. **Baishen Liang**: Conceptualization: Lead; Formal analysis: Lead; Methodology: Lead; Software: Lead; Supervision: Equal; Writing – review & editing: Lead. **Ruiming Wang**: Funding acquisition: Lead; Supervision: Supporting; Writing – review & editing: Supporting.

## DATA AND CODE AVAILABILITY STATEMENTS

All data and analysis code are available in an OSF repository: https://osf.io/v94hr/overview.

## Supplementary Material



## TECHNICAL TERMS

Syllables: Are basic units of the organization of speech sound sequences, containing vowels and usually also consonants.
Consonants: Are phonemes articulated by the obstruction of airflows from articulators in the mouth or larynx.
Lexical tones: Are speech cues that determine word meanings by pitch variation.
Phonemes: Are the smallest possible speech units that distinguish one word from another.
Vowels: Are phonemes produced without the obstruction of airflow from the vocal tract.
Transcranial alternating current stimulation: Is a kind of non-invasive brain stimulation that delivers sinusoidal alternating electric currents to the scalp.
Hierarchical drift-diffusion model (HDDM): Is a computational model that describes perceptual decision making as a process of evidence accumulation using parameters such as boundary threshold (*a*), drift rate (*v*), and starting point (*z*).
Psychometric curves: Are the sigmoid-shaped functions that map stimulus properties (e.g., pitch heights) to behavioral responses (e.g., tone levels) and measure perceptual parameters.
Neural oscillations: Are rhythmic and repetitive neural electrical activity that contain various frequency bands indexing different cognitive functions.
