# Supplementary material for: Theta and Gamma Transcranial Alternating Current Stimulation (tACS) Modulate Mandarin Consonant and Lexical Tone Perception
Source: Neurobiol Lang (Camb). 2026 Sep 14;7:NOL.a.285. doi: 10.1162/NOL.a.285 (PMC13612842; doi:10.1162/NOL.a.285)
Supplement: Supplementary file 1 [file nol-07-285-s001.docx]

**Supplementary Materials 1**

**Questionnaire 1. Transcranial Alternating Current Stimulation (tACS) Experiment Volunteer Screening Questionnaire**

[This questionnaire is intended to screen potential participants for a behavioral experiment. Only currently enrolled university students are eligible to complete this form.]

If you agree to participate in this experiment, please answer the following questions. The information you provide will be used solely for screening purposes and will be kept strictly confidential.

1. Do you have any neurological or psychiatric disorders? If yes, please provide details (e.g., type of disorder, duration, current medication status).
   ☐ Yes ☐ No
2. Do you currently have, or have you ever had, epilepsy, febrile seizures in childhood, or recurrent fainting episodes?
   ☐ Yes ☐ No
3. Has anyone in your immediate or extended family been diagnosed with epilepsy? If yes, please specify your relationship to the patient.
   ☐ Yes ☐ No
4. Have you ever undergone neurosurgery (including ophthalmologic surgery)? If yes, please provide details.
   ☐ Yes ☐ No
5. Do you currently have any of the following implanted medical devices?

- Cardiac pacemaker
- Cochlear implant
- Drug infusion pump
- Surgical clips

☐ Yes ☐ No

6. Are you currently taking any prescription or non-prescription medication? If yes, please provide details.
   ☐ Yes ☐ No
7. Are you currently experiencing dental pain or ear pain?
   ☐ Yes ☐ No
8. Are you currently pregnant, or do you suspect that you may be pregnant?
   ☐ Yes ☐ No
9. Can you think of any other reasons not mentioned above that would prevent you from participating in this study?
   ☐ Yes ☐ No

**Supplementary Materials 2**

**Questionnaire 2. Musical Training Experience Questionnaire**

1. Name:
2. Gender:
3. Age (please indicate in full years, as of the date of testing):
4. Date of Birth:
5. Education Level (please indicate your current highest educational attainment and total years of education).
    Example: Grade 8 in middle school, 8 years
6. Marital Status:
7. Do you have any neurological disorders? □ Yes □ No
8. Do you have any psychological disorders? □ Yes □ No
9. Have you ever received musical training? □ Yes □ No

**If you answered “Yes,” please continue with the following questions:**

1. At what age did you begin formal musical training?
    □ Before age 7 □ Age 8–12 □ Age 13–18 □ Age 18 or older
2. For how many consecutive years did you receive formal musical training?
    Total: ______ years
3. What was your primary area of specialization during formal music training?
    □ Vocal □ Orchestral (strings/winds/brass) □ Percussion □ Other: ______
4. How many instruments are you proficient in? Please specify the instrument(s), the approximate age you started, and the number of years studied.
    □ None □ 1 □ 2 □ 3 or more

 	Instrument: ______ Starting Age: ______ Years of Study: ______
 	Instrument: ______ Starting Age: ______ Years of Study: ______
 	Instrument: ______ Starting Age: ______ Years of Study: ______

5. How many instruments can you play but are not proficient in? Please specify the instrument(s), the approximate age you started, and the number of years studied.
    □ None □ 1–2 □ 3–4 □ More than 4

 	Instrument: ______ Starting Age: ______ Years of Study: ______
 	Instrument: ______ Starting Age: ______ Years of Study: ______
 	Instrument: ______ Starting Age: ______ Years of Study: ______
 	Instrument: ______ Starting Age: ______ Years of Study: ______

6. In the past three years, how frequently have you engaged in musical activities (on average per month)?
    Frequency: ______ times/month  Type(s): ______
7. On average, how long does each musical activity last?
    Duration: ______ hours

**Supplementary Materials 3**

**Questionnaire 3. Edinburgh Handedness Inventory^1^**

For each of the following tasks, please select the hand you **prefer** to use.

- If your preference is very strong, such that you would never use the other hand unless forced, please select **Left hand** or **Right hand**.
- If there is no clear preference and you use **both hands equally**, please select **Both hands**.
- Some activities naturally require both hands. In such cases, please judge only the hand preference for the object indicated in parentheses.

| **Task / Object** | **Left hand** | **Both hands** | **Right hand** |
| --- | --- | --- | --- |
| 1. Writing | ☐ | ☐ | ☐ |
| 2. Drawing | ☐ | ☐ | ☐ |
| 3. Throwing | ☐ | ☐ | ☐ |
| 4. Scissors | ☐ | ☐ | ☐ |
| 5. Toothbrush | ☐ | ☐ | ☐ |
| 6. Knife (without fork) | ☐ | ☐ | ☐ |
| 7. Spoon | ☐ | ☐ | ☐ |
| 8. Broom (upper hand) | ☐ | ☐ | ☐ |
| 9. Match (holding the match) | ☐ | ☐ | ☐ |
| 10. Opening a box (lid) | ☐ | ☐ | ☐ |

**Scoring:**

- Left hand = –2 points
- Both hands = 0 points
- Right hand = +2 points

**Calculation:**

- **LH =** total left-hand points
- **RH =** total right-hand points
- **CT =** LH + RH (cumulative total)
- **D =** RH – LH (difference score)
- **R =** (D / CT) × 100 (laterality quotient)

**Interpretation:**

- Left-handed: R < –40
- Ambidextrous: –40 ≤ R ≤ +40
- Right-handed: R > +40

*Reference:*
Oldfield, R. C. (1971). *The assessment and analysis of handedness: The Edinburgh Inventory*. Neuropsychologia, 9, 97–113.

**Supplementary Materials 4**

**Questionnaire 4. Post-Stimulation Sensation Questionnaire**

1. **Participant ID** (Format: Serial number + Initials in uppercase. Example: N01XXX. The serial number will be provided by the experimenter.)
2. **Session Number:**
    ☐ Pilot Session
    ☐ Formal Session 1
    ☐ Formal Session 2
    ☐ Formal Session 3
3. During the experiment, did you experience any of the following symptoms? Please rate **only symptoms that are related to electrical stimulation** (e.g., if a headache was due to fatigue and not stimulation, do not assign a high score).

|  | **Scale:** 1 = None 2 = Mild 3 = Moderate 4 = Severe |
| --- | --- |
| Headache |  |
| Neck pain |  |
| Scalp pain |  |
| Scalp injury |  |
| Skin redness / heating sensation |  |

4. During the experiment, did you experience any of the following symptoms? Please rate **only symptoms that are related to electrical stimulation** (e.g., if sleepiness was due to fatigue and not stimulation, do not assign a high score).

|  | **Scale:** 1 = None 2 = Mild 3 = Moderate 4 = Severe |
| --- | --- |
| Sleepiness |  |
| Difficulty concentrating |  |
| Sudden mood changes |  |
| Dizziness |  |

5. During the experiment, did you experience any of the following visual symptoms? Please rate **only symptoms that are related to electrical stimulation** (e.g., if blurred vision was due to fatigue and not stimulation, do not assign a high score).

|  | **Scale:** 1 = None 2 = Mild 3 = Moderate 4 = Severe |
| --- | --- |
| Phosphenes (perception of flashing lights) |  |
| Blurred vision |  |

6. During the experiment, did you experience the following symptom?

|  | **Scale:** 1 = None 2 = Mild 3 = Moderate 4 = Severe |
| --- | --- |
| Sensation of increased weight or pressure around the electrode sites or on the head |  |

7. **Other (please specify):** _____________________________

**Supplementary Materials 5**

**Fig S1. Points of subjective equity (**PSEs) of the psychometric functions for consonant and tone identification tasks under theta and gamma tACS in quiet and noise conditions. Bars indicate group means; error bars indicate SEM. No significant PSE effects were found (*p*s > 0.05).
